# Single-Cell Transcriptome Reveals Cell Type–Specific Molecular Pathology in a 2VO Cerebral Ischemic Mouse Model

**DOI:** 10.1007/s12035-023-03755-4

**Published:** 2024-01-05

**Authors:** Qian Zhang, Zhong Xu, Jian-Feng Guo, Shang-Hang Shen

**Affiliations:** The First Affiliated Hospital of Xiamen University, Medical College of Xiamen University, Xiamen, 361003 China

**Keywords:** Cell types, Ischemia-induced memory impairment, Single-cell sequencing, Two-vessel occlusion ischemia

## Abstract

Post-ischemia memory impairment is a major sequela in cerebral ischemia patients. However, cell type–specific molecular pathology in the hippocampus after ischemia is poorly understood. In this study, we adopted a mouse two-vessel occlusion ischemia model (2VO model) to mimic cerebral ischemia–induced memory impairment and investigated the single-cell transcriptome in the hippocampi in 2VO mice. A total of 27,069 cells were corresponding 14 cell types with neuronal, glial, and vascular lineages. We next analyzed cell-specific gene alterations in 2VO mice and the function of these cell-specific genes. Differential expression analysis identified cell type–specific genes with altered expression in neurons, astrocytes, microglia, and oligodendrocytes in 2VO mice. Notably, four subtypes of oligodendrocyte precursor cells with distinct differentiation pathways were suggested. Taken together, this is the first single-cell transcriptome analysis of gene expression in a 2VO model. Furthermore, we suggested new types of oligodendrocyte precursor cells with angiogenesis and neuroprotective potential, which might offer opportunities to identify new avenues of research and novel targets for ischemia treatment.

**Supplementary Information:**

The online version contains supplementary material available at 10.1007/s12035-023-03755-4.

## Introduction

Stroke, the second leading cause of death in the world [1, 2] and the leading reason for death in China [3], not only results in great suffering for patients and family members, but also carries heavy financial burden for individuals and society. Despite an estimated cost of treating stroke needs > 70 billion dollars per year in the USA, the treatment method of stroke remains very limited and currently is mainly based on supportive therapies [4]. Thus, there is an urgent need to explore novel options of treatment in stroke.

It is well documented that many stroke survivors display a range of cognitive and psychiatric impairments such as depression [4, 5]. Indeed, more than 30% of patients may develop cognitive impairment after stroke [6]. Memory impairment is considered one of the main complications of ischemic stroke [7]. Therefore, novel therapeutic strategies to reduce the incidence of cognitive impairment by identifying risk factors in the affected brain regions are required.

Promoting nerve regeneration is considered one of the main treatment methods for neuronal damage following stroke [8], but cell types involved in neurogenesis have yet to be fully elucidated. Moreover, identification of the molecular mechanisms underlying ischemic brain damage and the findings for key signaling pathways and protein molecules are important to guide the clinical treatment of ischemia.

Differences between cells can be greater yet at the RNA level, even within seemingly uniform populations. Diverse cell types have unique transcriptomes, which can be used to assess the gene regulation network underlying their physiological functions, behavior, and phenotype. Although bulk transcriptomic studies in model organisms have been at the forefront of research [9, 10], the cell type–specific molecular pathology of ischemia is unclear. Single-cell transcriptome profiling can identify biologically relevant differences in cells, even when cells may not be distinguishable by marker genes or cell morphology, and can be used to group cells in an unbiased way. Differences in transcriptomes between distinct cells may also provide critical information on the composition of cell types in diseased tissues. To understand the basis and importance of heterogeneity and stochastic aspect of gene expression, it is essential to examine transcriptomes of individual cells. Thus, there is a need to elucidate how individual cell types are affected after ischemia and clarify if the process of repair follows a similar blueprint in all cell types or whether certain cell types have unique transcriptional changes.

In this study, to address these issues, we adopted a two-vessel occlusion (2VO) ischemia mouse model to mimic cerebral ischemia–induced memory impairment and used single-cell RNA sequencing to profile and compare the cellular composition and transcriptome of cells in hippocampi of sham and 2VO mice. We provided comprehensive datasets of genes and pathways where transcriptional profile changes occurred after 2VO surgery for all the major cell populations. Our findings suggest that oligodendrocyte precursor cells (OPCs) are multi-functional cells in the adult hippocampi of mice, that is, they can differentiate into different cell types, including neurons, astrocytes, and oligodendrocytes. Furthermore, the presence and quantification of individual cell populations at various stages of differentiation, from stem-like cell to progenitor cell, to immature and mature neuron, are clearly defined at single-cell resolution. Finally, we suggest that the subtypes of OPCs have the potentiation to differentiate into neuronal precursor cells (NPCs) in the hippocampi of 2VO mice. Together, these results provide a promising therapeutic strategy for the treatment of cerebral ischemia.

## Material and Methods

### Mouse 2VO Cerebral Ischemia Model

All procedures and 6–10-week-adult male mice used were approved by the Animal Care and Use Committee of Fujian Medical University. Conventional SPF C57BL/6 mice were purchased from Experimental Animals Center of Fujian Medical University (Fuzhou, Fujian, China). Mice were maintained in a pathogen-free SPFII animal facility in a condition-controlled room (23 ± 1 °C, 50% ± 10% humidity) under a 12-h light/dark cycle. Mice were housed in groups of four to five in ventilated cages and given access to food and water ad libitum unless otherwise stated in the methods below. Experimenters were blinded to the treatments and sample processing throughout the subsequent experiments and analyses.

Bilateral occlusion of the common carotid arteries was performed as previously reported [11]. This model gives typical reproducible brain damage and it does not require cannulation of both the internal jugular vein and the femoral artery to continuously monitor mean arterial blood pressure during ischemia. Briefly, before surgery, mice were provided water ad libitum. The mean arterial blood pressure was measured using a non-invasive blood pressure system (Product #CODA, Kent Scientific Corporation, Torrington, CT, USA). Mice were anesthetized with isoflurane (5% for induction and 1.5–2.5% for maintenance). Rectal temperature was maintained at 37.0 ± 0.5 °C during the entire surgical procedure by a rectal temperature probe and heating pad (Product # TCAT-2 Temperature Controller, Harvard Apparatus, Holliston, MA, USA). The ventral neck was shaved to expose the skin and then disinfected. Next, an incision in the midline of the ventral neck region was made with a scalpel, the superficial fascia was dissected to expose the bilateral common carotid arteries, and both carotid arteries were clamped with micro-vessel clamps for 50 min to induce global cerebral ischemia. Hippocampal blood flow was detected by using a laser Doppler flowmetry (moorVMSLDF2, Moor Instruments, Wilmington, DE, USA) with a 0.5-mm flexible fiber optic as previously described [12]. The isoflurane level was decreased to 0% approximately 2 min before the release of the micro-vessel clamps, when blood reperfusion resumed. After, the neck incision was sutured with 5–0 sterile silk sutures. Mice were moved to a warm (37.0 ± 1 °C) recovery chamber (Product #DW-1, Harvard Apparatus, Holliston, MA, USA) to prevent post-ischemic hypothermia. For sham operations, micro-vessel clamping was performed on the bilateral common carotid arteries. The clamping was immediately released to allow for instant reperfusion. The subsequent procedure is same to that performed on cerebral ischemia group, including anesthesia processes.

### Morris Water Maze

The Morris water maze was used to determine spatial learning ability and memory. The procedure was performed in a white-water pool (120 cm in diameter with 2/3 full of water) containing a circular bright black platform (14 cm in diameter and submerged 1.5 cm beneath the water surface). Various patterns were equally distributed around the wall as visual cues for mice during the experiment. The experiment was initiated at day 7 of 2VO surgery, at the training stage, where the spatial acquisition learning ability training consisted of five consecutive days with every training day comprising of four trials with a 15-min inter-trial interval. The entry points of the mice were randomly selected each time from different designated locations. Once a mouse successfully found the platform within 60 s, it was placed into a cage under a warming lamp as a reward. Otherwise, the mouse was gently and manually guided to the platform and allowed to remain there for at least 20 s. The escape latency and pathlength to the platform were recorded each day to assess the spatial learning ability.

On day 6 (day 12 after 2VO surgery), a probe trial test was performed. The hidden platform was removed from the pool, and mouse was placed in the quadrant diagonally opposite the target quadrant and allowed to swim for 60 s freely. The percentage of time spent in the target quadrant was recorded and analyzed as a measure of spatial memory retention. Each mouse was monitored by a camera, and its trajectory was analyzed using the Smart software (V3.0, Panlab Harvard Apparatus, Holliston, MA, USA).

### Cell Isolation

Single cells were obtained according to a previous procedure with brief modifications [13]. Briefly, the individual adult male mouse was deeply anesthetized in an isoflurane chamber and decapitated. The brain was removed and rapidly dissected the whole hippocampus. The tissue was then cut thoroughly into pieces and transferred to a 1.5-mL microcentrifuge tube with 2 mg/mL isolation solution, containing pronase (Sigma, Cat# P6911-1G, Burlington, MA, USA) and 50 μg/mL DNaseI (Sigma, Cat# D5025, Burlington, MA, USA) in 1 mL Hibernate A (Invitrogen, Cat# A1247501, Waltham, MA USA)/B27 (Invitrogen, Cat# 17504, Waltham, MA USA) medium (HABG). The tissue and solution were mixed for 30 min at 34 ℃ in a horizontal shaker at 200 rpm. After incubation, the tissue was gently triturated by polished tips, and the single cells were released. The purified cells were obtained by density gradient centrifugation at 800 g for 15 min and resuspended in PBS (calcium and magnesium-free) containing 1% BSA, then centrifuged at 200 g for 2 min. The single cells were concentrated and resuspended in the desired medium. Cell number was determined and viability was measured by using trypan blue staining.

### Single-Cell RNA Sequencing and Raw Data Preprocessing

Cell numbers and viability of single-cell suspensions were determined, and samples with cell survival rates above 80% were applied for subsequent procedures. The cells were washed and resuspended to prepare a suitable cell density of 700–1200 cells/μL for 10 × Genomics Chromium ™ system operation (Pleasanton, CA, USA), without using antibodies or fluorescence-activated cell sorting for specific cell sorting, in order to reflect the original true cell type and proportion as much as possible. Based on the expected number of target cells, gel bead in emulsions (GEMs) were constructed for single-cell isolation. After the GEMs were formed, they were collected and reverse-transcribed in a PCR machine to achieve labeling followed the manufacturer’s instructions of 10 × Genomics. Next, the GEMs were destroyed; the first strand cDNA was purified and enriched with magnetic beads and subjected to cDNA amplification and quality control. Qualified cDNAs were applied for library construction using the 10 × Genomics Single Cell 3′ v2 Reagent Kit, and then fragmentation, adding adapters, and PCR amplification of the cDNA followed the manufacturer’s instructions. Finally, the Illumina NovaSeq platform with the PE150 sequencing mode was used for sequencing with a sequencing volume of > 50 k reads/cell.

Sequenced samples were processed using the Cell Ranger 3.1.0 pipeline and aligned to the GRCm38/mm10 mouse reference genome. Cell with fewer than 650 detected genes/cell and genes that were expressed by fewer than 20 cells (0.025% of all cells in the dataset) were removed before cell centering, scaling and identification of variable genes in the dataset.

### Dimensionality Reduction and Clustering

To visualize and interpret single-cell RNA sequencing data, two-dimensional projections of cell populations the dimensionality of the gene expression matrix was determined using principal component analysis (PCA). Next, to further reduce the dimensionality of these components, t-distributed stochastic neighbor embedding (t-SNE) [14] and uniform manifold approximation and projection (UMAP) [15] were employed. As tSNE is a nonlinear embedding that does not preserve distances, we used UMAP to interpret the distances of clusters and cell types in this study.

### Determination of Cell-Type Identity

We use two methods to resolve the identities of the cell clusters. First, known cell type–specific markers from previous studies were curated and checked for expression patterns in the cell clusters. A cluster showing high expression levels of a known marker gene specific for a particular cell type was considered to carry the identity of that cell type. Second, we evaluated the overlap between known marker genes of various cell types with the marker genes identified in our cell clusters. Overlap was assessed using a Fisher exact test and significance was set to Bonferroni-corrected *P* < 0.05. A cluster was considered to carry the identity of a cell type if the cluster marker genes showed significant overlap with known markers of that cell type. The two methods showed consistency in cell identity determination. Known markers for major hippocampal cell types have been previously described in the literatures. These include, but are not limited to, *Pdgfra* for oligodendrocyte precursor cells [16], *Cldn11* for mature oligodendrocytes [16], *Gjb6* for astrocytes [17], *Sox11* for neural progenitor cells [18], *Slc17a7* for glutamatergic neurons [19], *Slc32a1* for GABAergic neurons [20], *Ccdc153* for ependymocytes [21], *Folr1* for choroid plexus epithelial cells [22], *Cldn5* for endothelial cells [23], *Kcnj8* for pericytes [23], *Acta2* for vascular smooth muscle cells [23], *Slc6a13* for vascular leptomeningeal cells [16], *Tmem119* for microglia [19], and *Pf4* for macrophages [24]. For this study, these markers were sufficient to define all major cell types.

### Significantly Dysregulated Gene Analysis

To find differentially expressed genes, we used the Mann–Whitney *U* test [25], a non-parametric test that detects differences in the level of gene expression between two populations. By using the Mann–Whitney *U* test, we compared the distributions of expression levels of every gene separately. *P* values were adjusted using the Bonferroni correction for multiple testing. Gene expression fold changes were calculated as Median (2VO)/Median (Sham). Significantly dysregulated genes were screened using the criteria of an absolute value of Foldchange > 1.5 and adjusted *P* value < 0.05.

### GSEA Hierarchical Clustering Analysis of Common Hallmark Pathways

To identify common hallmark pathways in cell types, we first applied GSEA enrichment analysis for all genes in the corresponding populations. Hallmark gene sets are coherently expressed signatures derived by aggregating many MSigDB [26] (Molecular Signatures Database) gene sets to represent well-defined biological states or processes. After each hallmark pathways were acquired, the negative normalized enrichment score (NES) was applied for hierarchical clustering and heatmap construction for significantly enriched pathways.

### Ligand-Receptor Interaction Analysis

The ligand-receptor interaction analysis was mainly based on CellphoneDB [27], a novel repository of ligands, receptors, and their interactions [28]. For cluster 6 (C6, hereafter), C10, C14, C15, C16, C19, C20, C23, and C24–C12 interactions and dysregulated ligands, receptors, and interactions with C12 from C6, C10, C14, C15, C16, C19, C20, C23, and C24 were extracted. Extracted ligands-receptors were shown with Cytoscape v3.6.1.

### Immunostaining

Mice were anesthetized with isoflurane and transcardially perfused with 0.1 M phosphate-buffered saline (PBS) followed by 4% paraformaldehyde (PFA) in PBS at seventh day of 2VO surgery. Brains were post-fixed overnight in PFA at 4℃. Each brain was then dissected and further fixed in 4% PFA for an additional 24 h, and then transferred to 15% sucrose in PBS followed by 30% sucrose until saturated. The brain was embedded in Tissue-Tek OCT compound, frozen in liquid nitrogen, and stored at − 80℃ before being cut into 20-μm coronal sections in a cryostat at − 20℃ (CM3050S, Leica). Free-floating sections were washed with PBS. For immunostaining, sections were incubated with blocking buffer (5% normal goat serum and 0.3% Triton X-100 in PBS) for 1 h at room temperature, and incubated with primary antibody (SMI-32, 1:300, NE1023, Calbiochem, USA; MBP, 1:500, ab40390, Abcam, UK) overnight at 4℃. Sections were washed in PBS and then incubated with the appropriate secondary antibody for 2 h at room temperature. Sections were washed again for 3 × 10 min in PBS. After washing, they were mounted on coverslips using Fluoroshield mounting medium with DAPI (ab104139, Abcam, Cambridge, UK) for image collection.

### Data Availability

The single-cell RNA sequencing data are available through NCBI’s Gene Expression Omnibus (https://www.ncbi.nlm.nih.gov/geo/) under the accession number GSE 171393.

### Statistical Analysis

GraphPad Prism 8 was used for data processing and analysis. All data were expressed as means ± SEM. We used the unpaired *t*-test, paired *t*-test, one-way analysis of variance (1-way ANOVA, with Tukey’s test was used for post hoc comparison), or 2-way ANOVA or 2-way repeated measured ANOVA to conduct data analysis with Bonferroni’s test or Sidak’s test was used for post hoc comparison. All data are presented as the mean ± SEM in all cases. Statistical significance was taken as *P* < 0.05.

## Results

### Model Validation and Behavioral Test

**Fig. 1:**
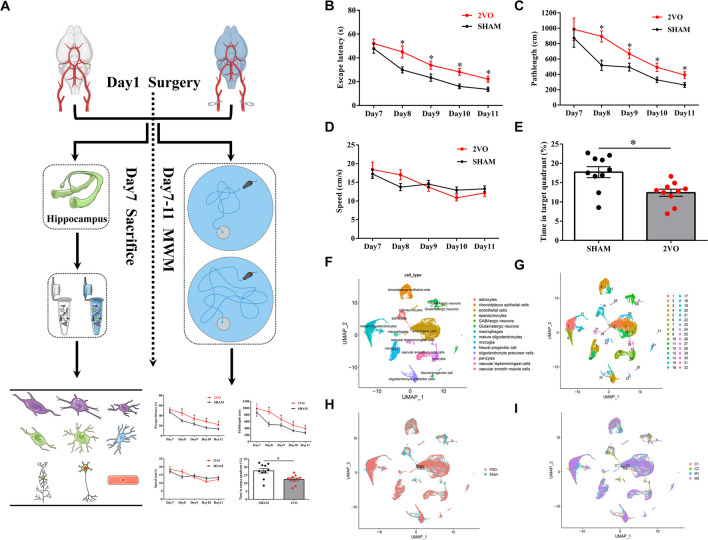
Single-cell atlas of 2VO cerebral ischemia model mice in the hippocampus. (**A**) Workflow of this study. (**B**) The Morris water maze test was performed during the indicated days. Escape latency to the platform (**B**) was measured during the acquisition test at 7, 8, 9, 10, and 11 days after 2VO surgery. Pathlength (**C**) and locomotion speed (**D**) were also recorded and plotted (sham: *n* = 10; 2VO: *n* = 10). (**E**) The percentage of time spent in the target quadrant during the spatial exploration stage of the Morris water maze probe test on the 12th day after surgery (sham: *n* = 10; 2VO: *n* = 10). Data are presented as the mean ± SEM. Error bars indicate SEM. * indicates a significant difference between the sham and 2VO groups; *P < 0.05; Repeated measured ANOVA with Bonferroni post hoc analysis was in (**B**)–(**D**); (**E**) Student’s *t*-test. (**F**) Thirteen cell types including neuronal, glial, and vascular cells of (**G**) thirty-two clusters were identified: clusters from (**G**) were color-coded corresponding to either (**H**) the two different types of animals analyzed (sham and 2VO) or (I) the four different batches that were sequenced

We firstly evaluated the pathological changes of myelin by SMI32 and MBP immunofluorescence double labeling (Supplementary Fig. 1). SMI32, an abnormally non-phosphorylated neurofilament protein, is a marker of axonal injury, whereas MBP is a marker of myelin integrity. The results showed that SMI32 was rarely expressed in the sham group, while the 2VO group showed increased expression of SMI32 and a loss of MBP intensity. After verifying the brain damage following surgery, we then examined whether 2VO cerebral ischemia mice displayed memory impairments in the Morris water maze test. The Morris water maze test was performed at days 7, 8, 9, 10, 11, and 12 after 2VO surgery. The results showed that 2VO cerebral ischemia mice presented significant memory decline 7 days after surgery compared to those in the sham group (Fig. 1B–E). In particular, the escape latency (time required to find the platform, in seconds) to the platform was higher during the acquisition test at days 7, 8, 9, 10, and 11 after 2VO surgery compared with that in the sham group (Fig. 1B, sham: *n* = 10; 2VO: *n* = 10; two-way RM ANOVA, interaction: *F*_(*4, 72*)_ = 0.7241, *P* = 0.5784; time: *F*_(*3.230, 58.14*)_ = 28.93, *P* < 0.0001; sham vs 2VO: *F*_(*1, 18*)_ = 38.57, *P* < 0.0001). Pathlength (distance covered by the mouse until it finds the platform, in centimeters) but not locomotion speed (velocity, in centimeters/second) was also increased (Fig. 1C and D, sham: *n* = 10; 2VO: *n* = 10; for pathlength: two-way RM ANOVA, interaction: *F*_(*4, 72*)_ = 0.9472, *P* = 0.4419; time: *F*_(*2.096, 37.74*)_ = 19.58, *P* < 0.0001; sham vs 2VO: *F*_(*1, 18*)_ = 16.14, *P* = 0.0008; for locomotion speed: two-way RM ANOVA, interaction: *F*_(*4, 72*)_ = 1.821, *P* = 0.1342; time: *F*_(*2.990, 58.82*)_ = 8.744, *P* < 0.0001; sham vs 2VO: *F*_(*1, 18*)_ = 0.01777, *P* = 0.8954). In addition, the percentage of time spent in the target quadrant during the spatial exploration stage of the Morris water maze probe test was decreased significantly (Fig. 1E, sham: *n* = 10; 2VO: *n* = 10; unpaired *t*-test, *F*_(*9, 9*)_ = 2.390, *P* = 0.053). All these results suggested that learning and memory was impaired after 2VO surgery.

### Single-Cell Atlas of 2VO Mice

**Fig. 2:**
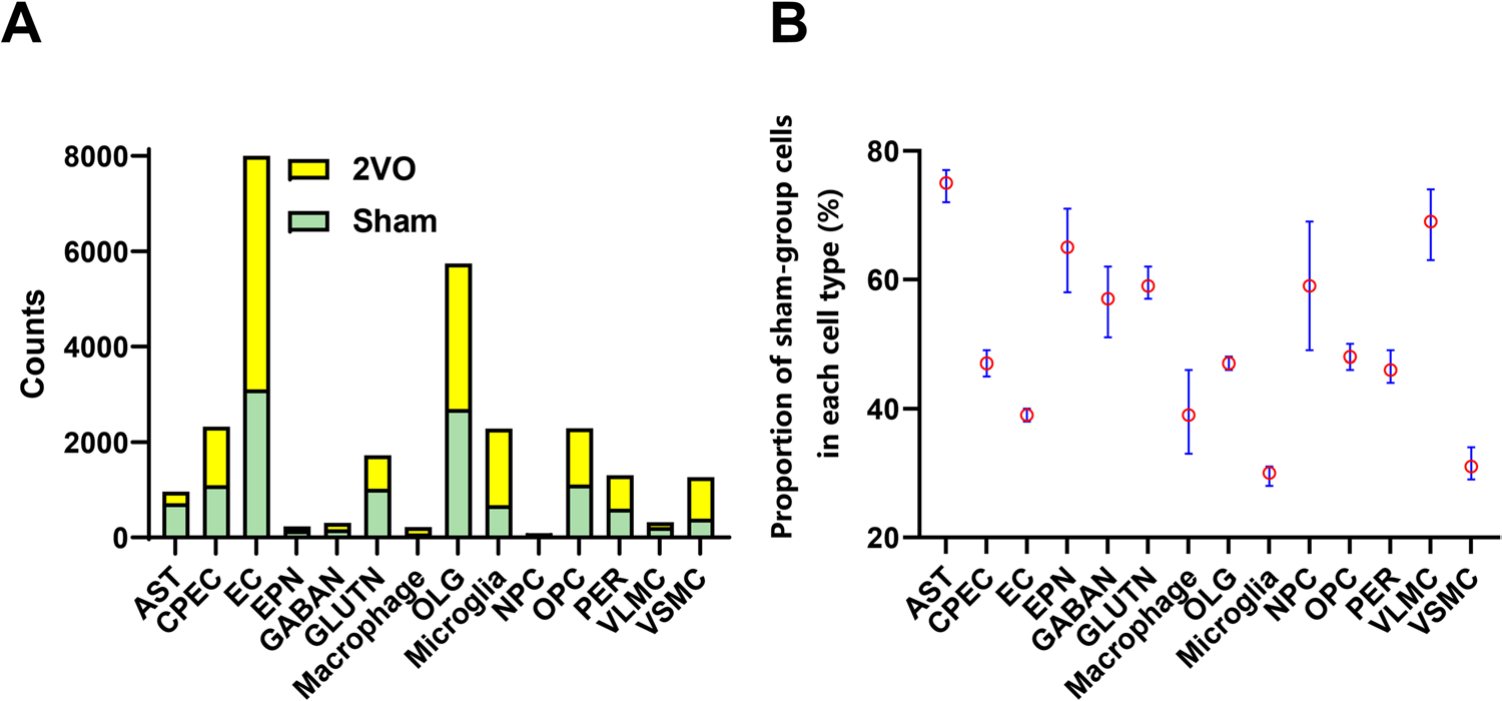
Results of chi-square test for RxC contingency. (**A**) Bar plots of cell counts in Sham and 2VO groups of different cell types. (**B**) The results of the RxC analysis of 95% CI for proportion of sham group in each cell type

Given that the hippocampus plays critical roles in learning and memory [29, 30], we used the 10 × Genomics platform to process high-throughput single-cell RNA sequencing to examine the transcriptional profiles of cells in the hippocampi from two sham and two 2VO ischemia model mice (Fig. 1A). After filtering, we generated 27,069 single-cell gene expression profiles, 12,018 from sham and 14,961 from 2VO model mice (Supplementary Table 1) The “Epitools” (https://epitools.ausvet.com.au/chisq) was employed to check cell numbers and proportions of each type by the chi-squared test for 2xN contingency tables with the CI95 ranges (Fig. 2 and Supplementary Tables 20–22). Of note, we found that the chi-square statistics was 1089.7 with the *P* value < 0.0001. And several cell types significantly deviated from 0.5 values and biased by our sampling procedure based on RxC analysis. Such as proportions of astrocytes (95% confidence interval (CI): 0.75 [0.72–0.77]), ependymocytes (95% CI: 0.65 [0.58–0.71]), and vascular leptomeningeal cells (95% CI: 0.69 [0.63–0.74]) in sham group were remarkably increased while endothelial cells (95% CI: 0.39 [0.38–0.40]), microglia (95% CI: 0.30 [0.28–0.31]), and vascular smooth muscle cells (95% CI: 0.31 [0.29–0.34]) were sharply decreased. Data were projected onto two dimensions via UMAP [15] to analyze cellular heterogeneity. In total, 32 clusters were obtained (Fig. 1G) and then we annotated clusters according to expression of known cell type markers (Supplementary Table 2) and identified 14 cell types, including OPCs, mature oligodendrocytes (OLGs), NPCs, astrocytes (ASTs), glutamatergic neurons (GLUTNs), GABAergic neurons (GABANs), ependymocytes (EPNs), choroid plexus epithelial cells (CPECs), endothelial cells (ECs), pericytes (PERs), vascular smooth muscle cells (VSMCs), vacuolar leptomeningeal cells (VLMCs), microglia, and macrophages (Fig. 1F). Clustering was not driven by experimental batch or individual samples (Fig. 1H and I), each mouse contains all clusters, and no cluster is unique to any mouse. Beyond retrieving known cell markers, we also identified possible marker genes for each hippocampal cell type, such as *Plpp3* for ASTs, *Cnp* for mature OLGs, *Birc5* for NPCs, and *Lhfpl3* for OPCs.

### Identification of Neuronal Characteristics After 2VO

**Fig. 3:**
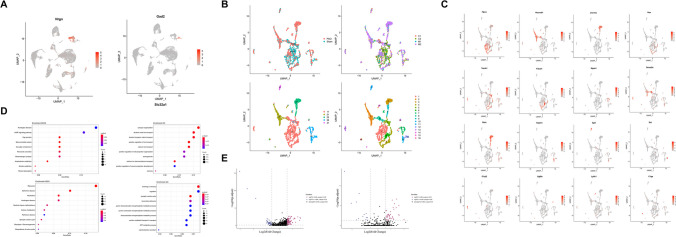
Identification of the neuronal characteristics after 2VO. (**A**) A representative possible marker gene of GLUTNs (left) and a representative possible marker gene of GABANs (right). (**B**) Neuronal clusters (lower-left) were reclustered into 16 subclusters (lower-right) corresponding to either the two different types of animals analyzed (upper-left, sham and 2VO) or the four different batches that were sequenced in neurons (upper-right). (**C**) Representative new marker genes of the 16 subclusters of neurons. (**D**) KEGG and GO dot plots of GABANs (upper) and GABANs (lower). (**E**) DEG analysis of sham and 2VO in glutamatergic (left) and GABAergic neurons (right)

Excitation and inhibition are the two basic characteristics of different types of neurons, which utilize glutamate and GABA as the major neurotransmitters for excitatory and inhibitory signals, respectively. To evaluate the features of different cell types in 2VO mice, we investigated the characteristics of two types of neurons in the hippocampus after 2VO injury. One thousand nine hundred thirty-four neurons (GLUTNs and GABANs with possible marker genes such as *Nrgn* for GLUTNs and *Gad2* for GABANs, Fig. 3A and Supplementary Table 3) were assembled into five clusters (Figs. 1G and 3B in lower-left). The clusters of the data enabled us to highlight more subtle changes within neurons. This secondary analysis identified 16 different subclusters (Fig. 3B, lower-right) with different possible marker genes (Fig. 3C and Supplementary Table 4). This process allowed us to generate a comprehensive dataset of gene expression profiles for all the experimentally validated cell subclusters from both sham and 2VO injured mice at high resolution. It also permitted us to identify specific markers that distinguish each subcluster regardless of 2VO treatment (Supplementary Table 4).

Kyoto Encyclopedia of Genes and Genomes (KEGG) and gene ontology (GO) analyses (Fig. 3D, upper) showed that differentially expressed genes (DEGs) of GLUTNs mainly belonged to the cAMP signaling and synaptic organization pathways, both of which are important in learning and memory [31, 32]. Of note, more than 30% (3/9: *Atp1b1*, *Atp2b1*, and *Atp2b2*) of DEGs in the cAMP signaling pathway belonged to the ATPase plasma membrane Ca^2+^ transporting family. We found that the expression of *Atp1b1*, *Atp2b1*, and *Atp2b2* decreased after 2VO surgery. Interestingly, *C1qb*, which participates in the organization of the synapse and is mainly expressed in microglia, was increased almost fourfold in neurons after 2VO surgery (from 0.345 to 1.359). *C1qb* is one of the complement components and associated with various diseases, such as neuropathic pain [33], brain lesion [34], and sporadic amyotrophic lateral sclerosis [35]. Our results showed that C1qb was upregulated in GLUTNs but not microglia after 2VO surgery, indicating its specific roles in the regulation of learning and memory in neurons.

In GABANs, after characterizing by using KEGG, the results (Fig. 3D, lower-left) showed that the DEGs were mainly involved in the ribosome pathway. After characterizing by using gene ontology, the results (Fig. 3D, lower-right) showed that many DEGs were involved in the learning and memory-related pathways, including the synaptic vesicle cycle, learning and/or memory, and cognition. GABANs control the inhibitory neural circuit in the adult brain, which is responsible for a variety of pathophysiological processes including modulation of cortical and hippocampal neural circuitry and activity, cognitive function–related neural oscillations, and information integration and processing. Abnormalities in GABANs function have been associated with multiple neurodevelopmental and neurodegenerative disorders [36]. It is therefore reasonable to speculate that upregulation of these DEGs may increase the number and density of synapses in GABANs, thereby increasing the transmission of synaptic signals, leading to the memory dysfunction observed in 2VO surgery mice.

Among the DEGs of GLUTNs (Fig. 3E, left), *Lars2*, which encodes enzymes catalyzing the aminoacylation of tRNA^Leu^, was the most significantly decreased gene in the 2VO group (Supplementary Table 5). By contrast, *Reln*, which is an extracellular matrix serine protease involved in neuronal migration in the developing brain [37], was identified as the most significantly increased gene after 2VO surgery. Indeed, *Reln* knockout mice show increased susceptibility to ischemic brain injury [38]. Consistently, our result showed that *Reln* was increased after ischemia. We found that *Lars2* was also the most significantly decreased gene in GABANs after 2VO surgery (Fig. 3E, right and Supplementary Table 6), whereas *Cnr1* was the most significantly increased gene.

### Identification of Astrocytic Characteristics After 2VO

**Fig. 4:**
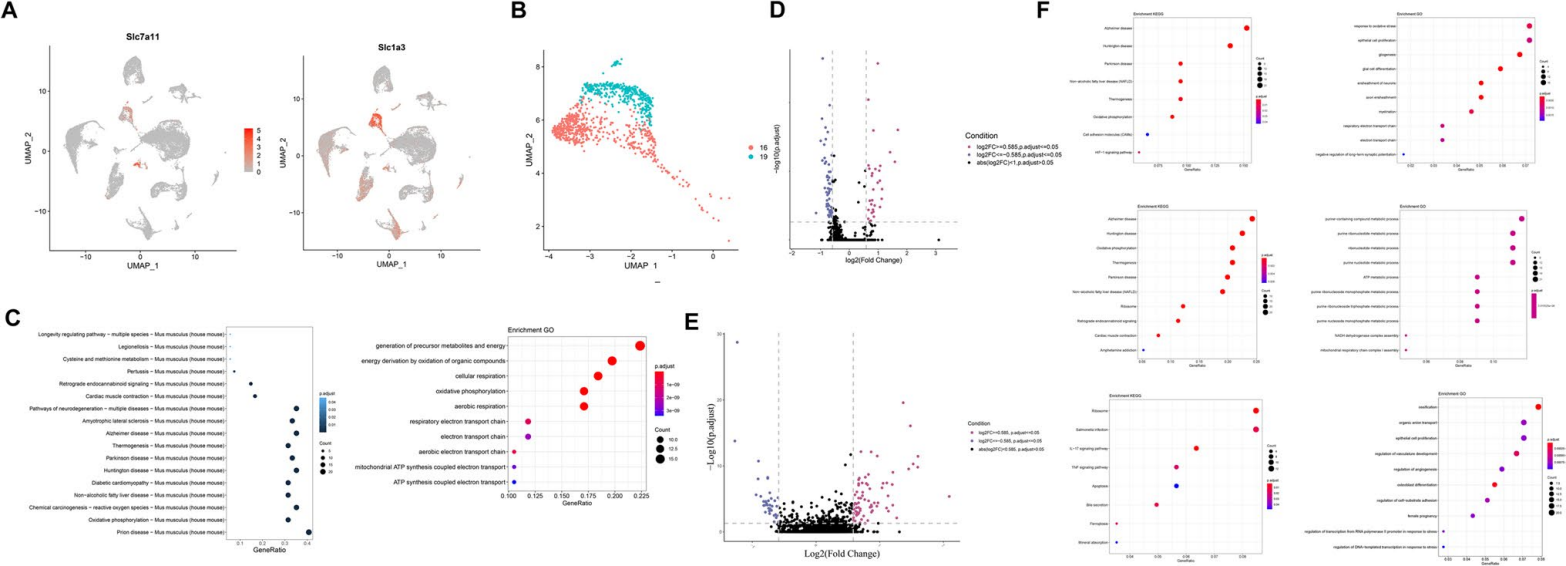
Identification of the astrocytic characteristics after 2VO. (**A**) Two represent possible marker genes of astrocytes. (**B**) UMAP visualization of the two clusters of astrocytes. (**C**) KEGG and GO dot plots of astrocytes. (**D**) DEGs analysis of sham and 2VO astrocytes. (**E**) KEGG and GO dot plots of cluster 16 (upper) and cluster 19 (lower) astrocytes

An increasing number of studies have begun to focus on the roles of ASTs in ischemia in recent years, but their role in the regulation of ischemia has not yet been detected at the single-cell level. To validate this, we investigated the function of ASTs in the hippocampus after 2VO surgery at the single-cell level. In the 32 clusters, using *Gjb6* as the known marker, we found that ASTs lineage included clusters 16 and 19 (Fig. 4B). We also identified possible marker genes for ASTs (Fig. 4A).

After characterizing by using KEGG, the results demonstrated that oxidative phosphorylation, degenerative diseases, and the HIF-1 signaling pathway were among the top dysregulated pathways (Fig. 4C, left and Supplementary Table 7). Most genes in these pathways belong to the cytochrome c oxidase family, including *Cox5a*, *Cox5b*, *Cox6c*, *Cox7c*, and *Cox8a*. Cytochrome c oxidase is the terminal enzyme of the mitochondrial respiratory chain [39]. Mitochondrial oxidative phosphorylation involves multi-enzyme complexes, including cytochrome c oxidase, which is located in the mitochondrial inner membrane [40]. A considerable part of cerebral ATP is consumed for neuronal electrogenic activity [41]. Regulatory mechanisms operate to ensure an adequate spatial and temporal energy supply by mitochondria, especially in ASTs, which is pivotal for neuronal excitability and survival. ASTs have emerged as active players in brain energy delivery, production, utilization, and storage. In our study, we found that all the cytochrome c oxidase family–related genes were down-regulated after 2VO surgery, indicating the possibility of energy insufficiency in neurons, leading to the impairment of memory. Indeed, after characterizing by using gene ontology, the (Fig. 4C, right) results also showed that many pathways related to energy metabolism were abnormal.

Interestingly, we found that several genes including *C1qa*, *C1qb*, and *Hexb*, which were mainly expressed in microglia [42, 43], were upregulated in astrocytes (Fig. 4D). Consistent with our results, *C1q* mRNA has also been reported to be dramatically upregulated in the brain after global ischemia and focal ischemic insult in rodents [44]. Recent research found that *Hexb* was persistently expressed by microglia and much less by central nervous system–associated macrophages in various disease models.

At last, we determined the differences in the two clusters of ASTs using KEGG and GO pathway analyses (Fig. 4E). The results showed that pathways of cluster 16 were different from cluster 19. Cluster 19 was focused on the metabolic process signaling pathways while cluster 16 was involved in the ensheathment of neurons. Taken together, the critical involvement of ASTs in normal brain function and their response to ischemic lesions designate them as excellent therapeutic targets for improving the functional prognosis after ischemia.

### Identification of the OPC Characteristics After 2VO Surgery

**Fig. 5:**
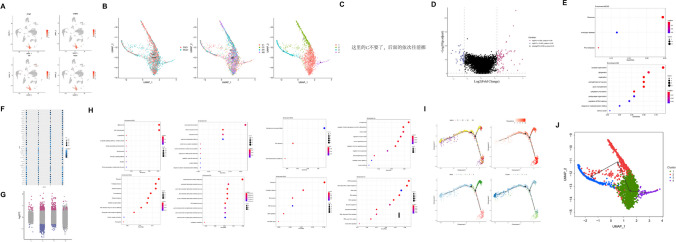
Identification of the OPC characteristics after 2VO. (**A**) Representative possible marker genes of OPCs. (**B**) UMAP visualization of OPCs: the four clusters of OPCs (right) are color-coded corresponding to either (left) the two different types of animals analyzed (sham and 2VO) or (center) the four different batches that were sequenced. (**C**) DEG analysis of sham and 2VO OPCs. (**D**) KEGG and GO dot plots of OPCs. (**E**) Dot plots of OPCs showing the expression level of the ten highest expressed genes per cluster. (**F**) DEG analysis of sham and 2VO OPCs in each cluster. (**G**) The KEGG and GO dot plots of cluster 1 (upper-left), cluster 2 (upper-right), cluster 3 (lower-left), and cluster 4 (lower-right) OPCs. (**H**) Pseudotime trajectory of OPCs, colored by different batches (upper-left), pseudotimes (upper-right), states (lower-left), and clusters (lower-right). (**I**) Slingshot analysis showing the differentiation route within the four OPC subtypes

Adult OPCs can be activated and proliferate following certain forms of central nervous system (CNS) damage, such as mechanical injury, excitotoxicity, and viral infection [45]. However, whether all OPCs have the same characteristics or functions are still largely not understood. We therefore investigated the function of OPCs in the hippocampus after 2VO surgery at the single-cell level. First, we identified possible marker genes for OPCs (Fig. 5A), such as *C1ql1*, *Lhfpl3*, and *Vcan*. Next, we detected 2292 OPCs (Fig. 5B and Supplementary Table 1) that we assembled into four clusters, renamed as OPC-1, 2, 3, 4. Clustering was not driven by experimental batch or individual sample (Fig. 5B).

Intriguingly, the top five upregulated DEGs of OPCs were all mitochondria-related genes (Supplementary Table 9), suggesting that OPCs may be actively dividing. Indeed, OPCs are believed to be multipotential and can differentiate into other cell types including neurons [46–48]. In support of this theory, KEGG and GO analyses were performed and the results showed that many genes were involved in myelination, the ensheathment of neurons, and gliogenesis (Fig. 5D and Supplementary Table 10). To further verify the functional differences of the four subclusters, we analyzed the DEGs of each cluster. The top ten expression genes of the four clusters are shown in Fig. 5E and the associated DEGs visualized in Fig. 5F. Most of the DEGs were present in cluster 2 and cluster 3. To validate whether DEGs of each cluster were enriched in different pathways, KEGG and GO analyses were employed to cluster them into distinct regulated pathways (Fig. 5G). OPC-1 was mainly enriched in the developmental maturation pathway, OPC-2 in neuron-related pathways such as axonogenesis and neuronal death pathways, OPC-3 in energy-related pathways, such as oxidative phosphorylation and ATP metabolic process pathways, and OPC-4 in cell proliferation–related pathways, such as DNA replication, RNA splicing, ribosome biogenesis, and RNA processing pathways. Together, all the data suggest that OPCs are very heterogeneous and that different subgroups of OPCs participate in various regulatory pathways, which might play diverse functions in ischemia. Given that the four clusters belong to the OPCs lineage, we then wanted to know the pseudotime trajectory inside the OPCs with Monocle. The pseudotime trajectory results are shown in Fig. 5H. To further investigate cell trajectories during OPC development, we utilized Slingshot [49], a statistical framework for inferring branching lineage assignments and developmental distances. The results showed that the developmental trajectory of OPCs was from OPC-4 to OPC-1, then to OPC-2 and finally to OPC-3 (Fig. 5I). The analysis also revealed that OPC-4 is most likely to be the multipotentiality cluster. For instance, *Pclaf* in OPC-4 may act as a regulator of DNA repair during DNA replication as it has been demonstrated to be crucial for cell proliferation [50, 51]. Notably, we found that cell-cycle gene signatures appeared in OPC-4 but not in the other three subclusters (Supplementary Table 11). Moreover, our UMAP clustering analysis showed the OPC-4 co-expressed 89.29% (100/112) of its high expression genes with cluster 30, namely NPCs. Taken together, our single-cell sequencing analysis suggest that OPCs, specifically OPC-4, may be multipotential cells and can differentiate into different types of cells in both the healthy and disease conditions in the hippocampus of adult mice.

### Identification of the EC Characteristics After 2VO

**Fig. 6:**
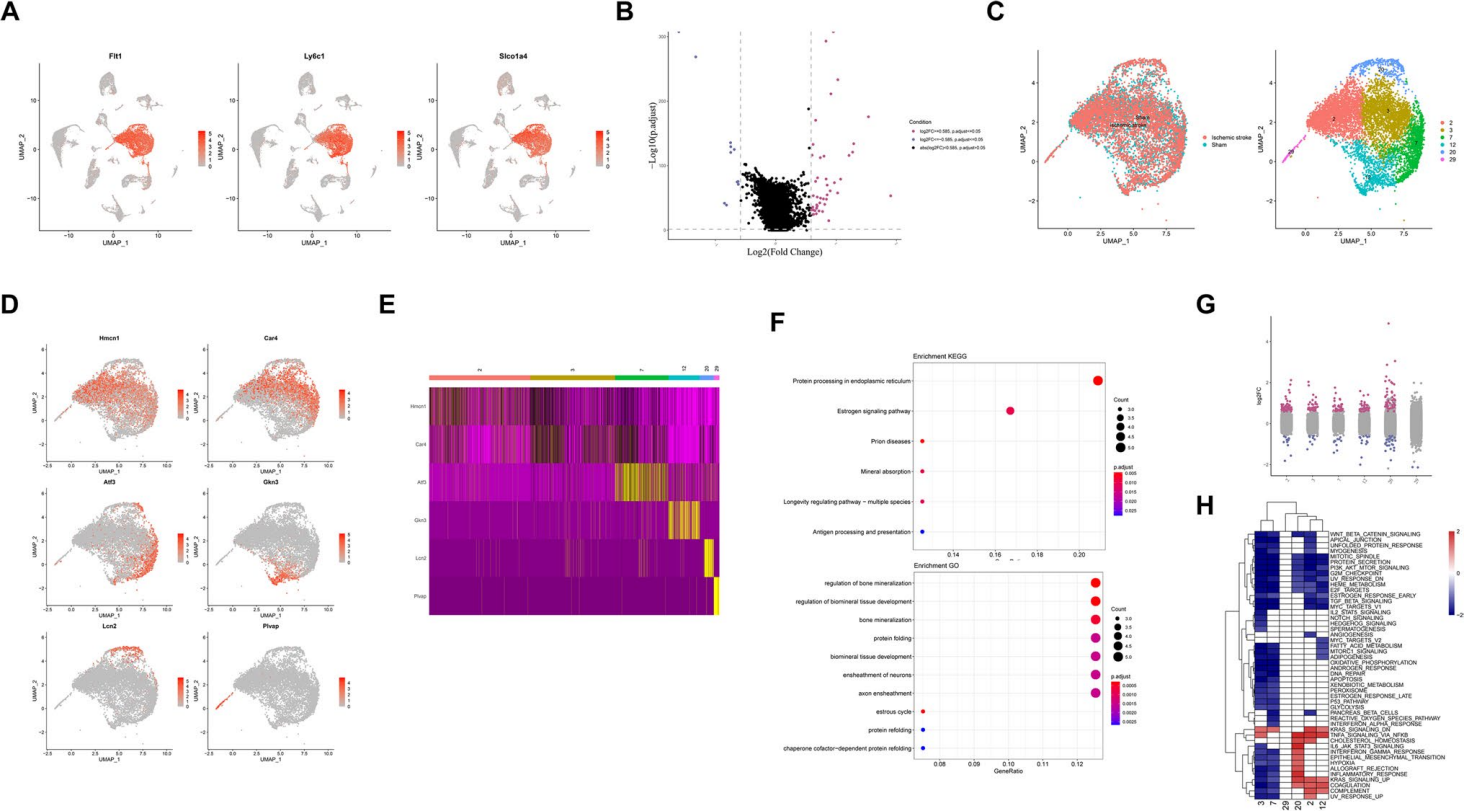
Identification of the EC characteristics after 2VO. (**A**) Representative possible marker genes of ECs. (**B**) DEG analysis of sham and 2VO ECs. (**C**) UMAP visualization of ECs: the six clusters of ECs (right) are color-coded corresponding to the two different types of animals analyzed (left, sham and 2VO). (**D**) Representative possible marker genes of the six clusters of ECs (*Hmcn1* enriched in cluster 2, *Car4* in cluster 3, *Atf3* in cluster 7, *Gkn3* in cluster 12, *Lcn2* in cluster 20, and *Plvap* in cluster 29). (**E**) Heatmap of ECs showing the expression levels of representative possible marker genes in each cluster. (**F**) KEGG and GO dot plots of ECs. (**G**) DEG analysis of sham and 2VO in each E cluster. (**H**) GSEA analysis based on hierarchical clustering pathways in the six EC subtypes

Vascular ECs are important in ischemia recovery [52]. Here, six clusters (cluster 2 (C2), C3, C7, C12, C20, and C29) with high *Cldn5* expression were identified as ECs (Fig. 6C). We also identified potential markers for ECs, such as *Flt1*, *Ly6c1*, and *Slco1a4* (Fig. 6A). Similar to the DEGs we found in GLUTNs (Fig. 3E, left), *Lars2* was also one of the most significantly decreased genes in ECs after 2VO surgery. The most upregulated DEG in ECs was *Ttr* (Supplementary Table 12). In addition, we found that *plp1*, a major component of mammalian CNS myelin with possible functions in myelin stability and maintenance, was sharply increased in ECs after ischemia (Supplementary Table 12).

After characterizing by using KEGG and gene ontology, the results showed that DEGs were mainly enriched in the protein processing pathway of the endoplasmic reticulum (Fig. 6F). To further clarify the functions of different subtypes of ECs, we analyzed the six subclusters of ECs to identify potential markers. Results indicated that *Hmcn1* was enriched in C2, *Car4* in C3, *Atf3* in C7, *Gkn3* in C12, *Lcn2* in C20, and *Plvap* in C29 (Fig. 6D and E). *Lcn2* has been previously used as one subcluster of brain vascular EC marker [53, 54] while other markers were proposed for the first time to identify different cell subsets of ECs. We next determined EC-specific DEGs (Fig. 6G), and conducted GSEA analysis for the six EC clusters (Fig. 6H). No DEGs were found in C29, suggesting that it is the most stable cluster of ECs while C20 accounted for the greatest number of DEGs. We found that 45.8% of DEGs in C3 and C7 were identical (22/48 and Supplementary Table 13), and most of the pathways in the GSEA analysis similar, suggesting they are closely related in cell lineage. Additionally, 40.0% of DEGs in C2 and C12 were identical (20/50 and Table 14); similarly, most of the pathways in the GSEA analysis were the same in C2 and C12, indicating that the two clusters are closely related in cell lineage. C20 was unique and possessed the most DEGs of all six EC clusters; therefore, we focused on EC20. As shown in Fig. 6H, enriched pathways in EC20 included the TNFα, cholesterol hemostasis, IL6-JAK-STAT3, IFN gamma, hypoxia, and inflammatory response, implying the cluster’s immune cell tendency. Indeed, after characterizing by using KEGG and gene ontology, the results showed that DEGs of cluster 20 were mainly enriched in antigen processing and presentation, IL-17 signaling and TNF signaling pathways (Supplementary Table 15).

### Identification of the Immune Cell Characteristics After 2VO

**Fig. 7:**
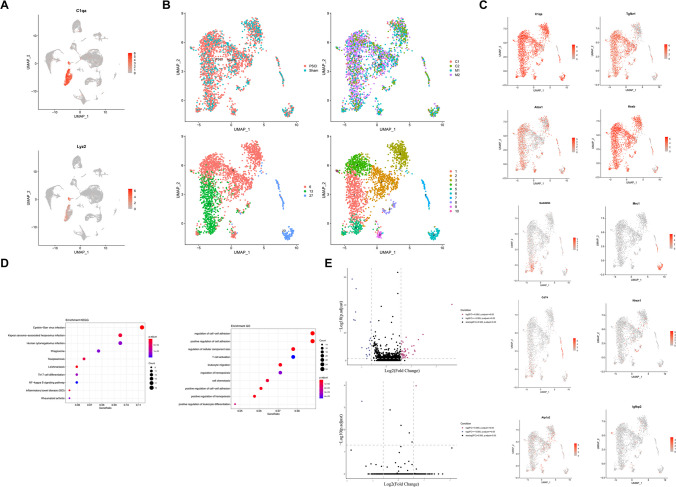
Identification of immune cell characteristics after 2VO. (**A**) Representative possible marker genes of immune cells (*C1qa* enriched in microglia and *Lyz2* in macrophages). (**B**) Immune cells (lower-left) were reclustered into ten subclusters (lower-right) corresponding to either the two different types of animals analyzed (upper-left, sham and 2VO) or the three different batches of sequencing in immune cells (upper-right). (**C**) Representative new marker genes of the ten subclusters of immune cells. (**D**) KEGG and GO dot plots of immune cells. (**E**) DEG analysis of sham and 2VO microglia (upper) and macrophages (lower)

We then investigated the characteristics of immune cells in the hippocampus after 2VO injury. In total, 2513 immune cells (including microglia and macrophage with possible marker genes such as *C1qa* for microglia and *Lyz2* for macrophages, Fig. 7A and Supplementary Table 16) were assembled into three clusters (Fig. 7B, lower-left). Further analysis identified ten different subclusters (Fig. 7B, lower-right) with different possible marker genes (Fig. 7C and Supplementary Table 17): *C1qa* was enriched in cluster 1, *Tgfrb1* in cluster 2, *Atox1* in cluster 3, *Hexb* in cluster 4, *Gadd45b* in cluster 5, *Mrc1* in cluster 6, *CD74* in cluster 7, *Nrxn1* in cluster 8, *Atp1a2* in cluster 9, and *Igfbp2* in cluster 10. After characterizing by using KEGG and gene ontology, the results showed that the DEGs of the immune cells were mainly enriched in the immune-related pathways including T cell activation, phagosome, and NF-kappa B signaling (Fig. 7D). *Apoe* is the most significantly increased DEG in microglia after 2VO surgery (Fig. 7E, upper).

### Identification of the OLG Characteristics After 2VO

**Fig. 8:**
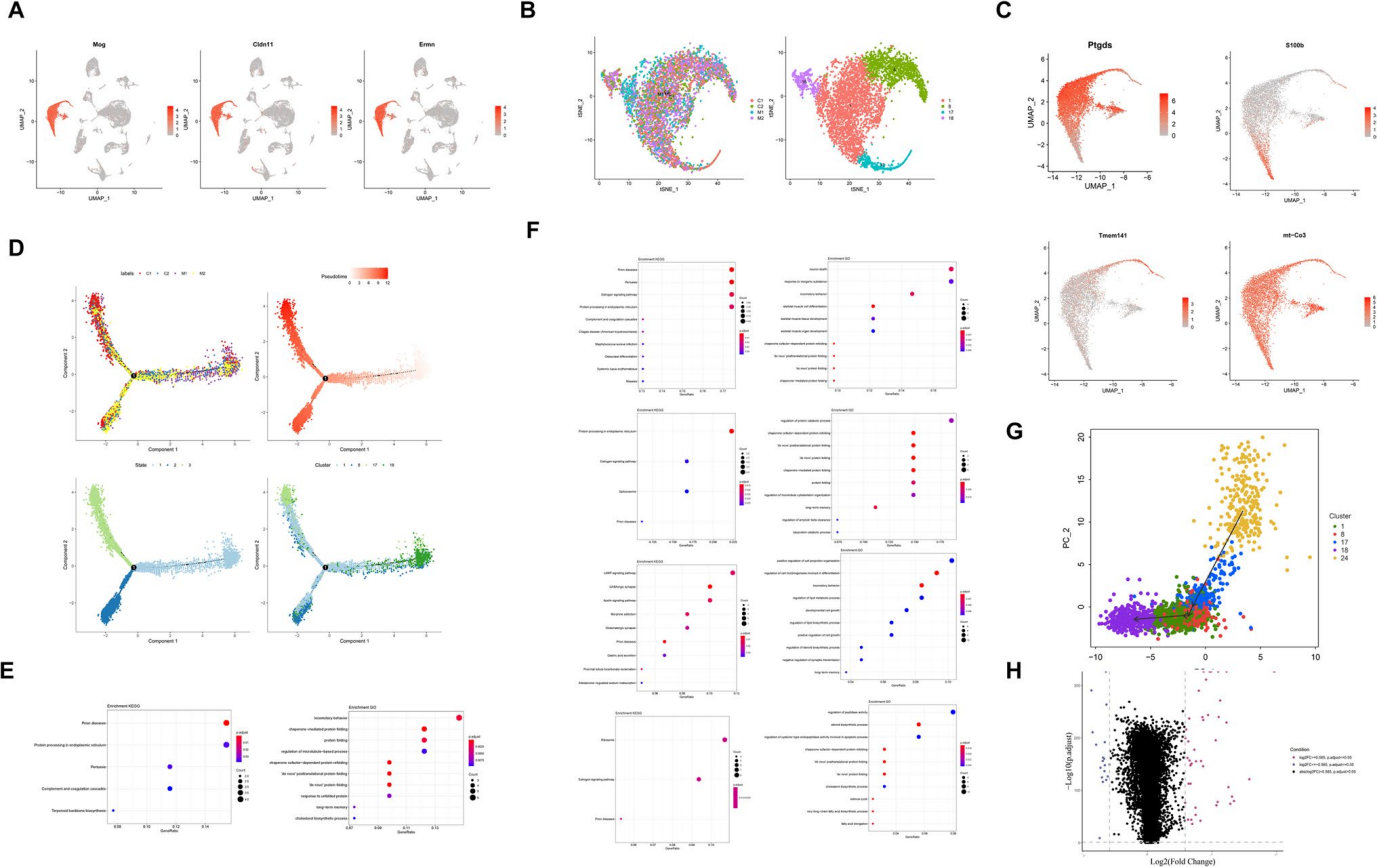
Identification of OLG characteristics after 2VO. (**A**) Representative possible marker genes of OLGs. (**B**) UMAP visualization of OLGs: the four clusters of OLGs (right) are color-coded corresponding to the two different types of animals analyzed (left, sham and 2VO). (**C**) Representative possible marker genes of the four clusters of OLGs (*Ptgds* enriched in cluster 1, *S100b* in cluster 8, *TMEM141* in cluster 17, and *mt-C03* in cluster 18). (**D**) Pseudotime trajectory of OLGs, colored by different batches (upper-left), pseudotimes (upper-right), states (lower-left), and clusters (lower-right). (**E**) KEGG and GO dot plots of OLGs. (**F**) KEGG and GO dot plots of cluster 1 (upper), cluster 8 (upper-center), cluster 17 (center-lower), and cluster 18 (lower) OLGs. (**G**) Slingshot analysis showing the differentiation route of the four OLG subtypes and cluster 24. (**H**) DEG analysis of sham and 2VOOLGs

To explore the functional changes of OLGs, we studied OLGs in the hippocampus after 2VO surgery at the single-cell level. First, we identified possible marker genes for OLGs (Fig. 8A), such as *Mog* and *Ermn*. We detected 5738 OLGs (Fig. 8B and Supplementary Table 1) that were assembled into four clusters. The potential possible markers of the four clusters are shown in Fig. 8C: *Ptgds* was enriched in cluster 1, *S100b* in cluster 8, *Tmem141* in cluster 17, and *mt-Co3* in cluster 18.

The pseudotime trajectory of OLGs is shown in Fig. 8D. Given that OLGs are derived from OPCs, and cluster 24 of OPCs was orientated to OLGs (Fig. 1G), we further investigated the developmental trajectory of cluster 24 and OLGs by Slingshot analysis. As expected, pseudotime analysis using Slingshot showed that the developmental trajectory order of OLGs was from cluster 24 of OPCs to cluster 17, cluster 8, then to cluster 1, and finally to cluster 18 (Fig. 8G). Although the DEGs of OLGs were mainly enriched in protein folding pathways in the GO analysis (Fig. 8E), we found that the DEGs of cluster 17 were enriched in cell growth and differentiation pathways (Fig. 8F), suggesting cluster 17 was upstream of other OLG clusters. And we found that *Neat1*was the most significantly upregulated DEG in OLGs after 2VO surgery while *Cnp* was the most significantly decreased DEG (Fig. 8H, Supplementary Table 18).

### Identification of Other Cell Type Characteristics After 2VO

**Fig. 9:**
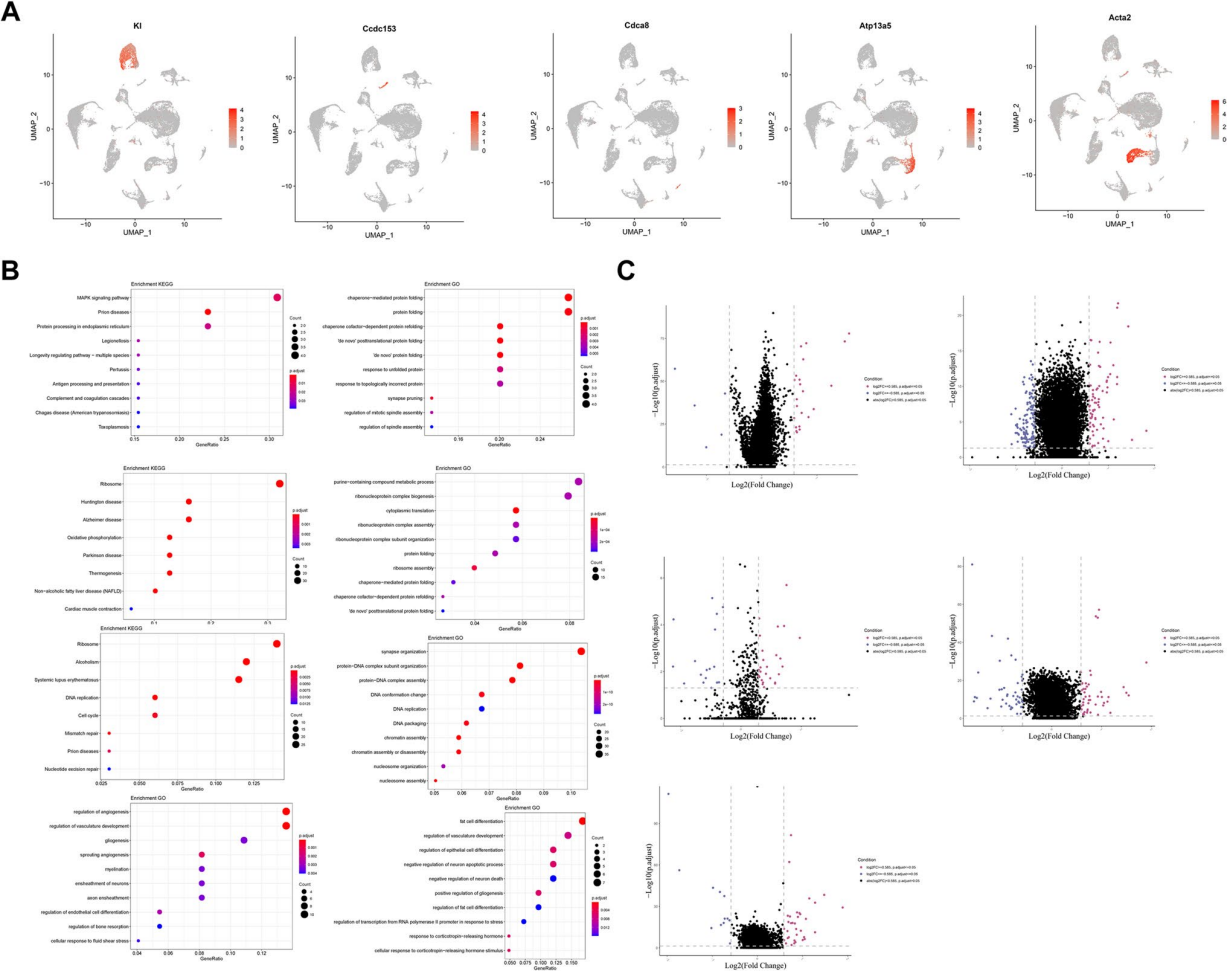
Identification of other cell type characteristics after 2VO. (**A**) Representative possible marker genes of different cell types (*KI* enriched in CPECs, *Ccdc153* in EPNs, *Cdca8* in NPCs, *Atp13a5* in PERs, and *Acta2* in VSMCs). (**B**) KEGG and GO dot plots of CPECs (upper), EPNs (center-upper), NPCs (center), PERs (center-lower), and VSMCs (lower). Please note that there was no KEGG enrichment analysis in PERs or VSMCs; therefore, not data is shown in the figure. (**C**) DEG analysis of sham and 2VO CPECs (upper-left), EPNs (upper-right), NPCs (left-center), PERs (right-center), and VSMCs (lower)

To explore the functional changes of other cell types, we examined the features of them including CPECs, EPNs, NPCs, PERs, and VSMCs in the hippocampus after 2VO surgery. First, we identified their potential possible markers: *Kl* was enriched in CPECs, *Ccdc153* in EPNs, *Cdca8* in NPCs, *Atp13a5* in PERs, and *Acta2* in VSMCs (Fig. 9A). KEGG analysis of CPEC showed that many of the enriched DEGS, including *Hspa1a*, *Hspa1b*, *Hspb1*, and *Dusp1* in the MAPK signaling pathway, were related with inflammatory modulation (Fig. 9B and C, upper-left).

After characterizing by using gene ontology, the results showed that the DEGs were largely enriched in protein synthesis–related pathways, including cytoplasmic translation, ribosome assembly, protein folding, and ribonucleoprotein complex assembly (Fig. 9B, center-upper); however, most of the DEGs were decreased (Fig. 9C, upper-right, Supplementary Table 19). Intriguingly, after characterizing by using KEGG and gene ontology, the results showed that the DEGs of PERs and VSMCs were mainly focused on DNA replication, chromatin assembly, vasculature development, and angiogenesis.

## Discussion

Here, we present a comprehensive single-cell resolution catalog of different cell types in the regulation of 2VO-induced memory impairment. By describing key molecular differences between various cell types, our analysis confirms many important previously reported observations obtained from in vitro studies, bulk experiments, and animal models, and highlights key areas for further study to advance our knowledge regarding the function of different cell types after post-ischemia memory impairment. By identifying novel cell subtypes and highlighting a catalog of marker genes, our research will fuel advances in post-ischemia memory impairment, and act as a method to remedy ischemia-induced memory impairment. Although we were unable to describe each subtype and its biological functions in detail, herein, some key observations are worthy of attention. First, the hippocampal environment between 2VO surgery and sham mice is more complex and heterogeneous than previously appreciated. Previous studies on post-ischemia memory impairment were mainly based on bulk sequencing. Here, we analyzed 27,069 cells by scRNA-seq and identified 14 cell types, including neurons, ASTs, immune cells, ECs, and others. Each subtype showed divergent pathway activities after 2VO surgery, suggesting that these cells function in distinct biological pathways.

Furthermore, 32 clusters were obtained. Although clustering was not driven by experimental batch or individual samples, each mouse contains all clusters, and no cluster is unique to any mouse (Fig. 1H and I); the cell proportions were indeed shifted after 2VO based on the RxC analysis results (*P* < 0.001). Because the dissociation of mammalian adult brains is challenging due to the complexity of the tissue, the estimated percentages for each cell type do not necessarily reflect their actual proportions in the mouse brain, mainly due to differences in their sensitivity to tissue dissociation. How to minimize this shift as much as possible is very valuable for truly understanding the differences after various treatments in the future. Proportions of 14 cell types were compared between the two groups, and the cell numbers of vascular ECs and OLGs were found to account for the majority. The cell counts of ASTs, neurons, and OLGs showed the most striking decrease, while the number of vascular endothelial and smooth muscle cells was increased after post-ischemia memory impairment. We also identified possible markers for each hippocampal cell type. We then investigated the cell type–specific molecular changes by performing DEG analysis. Interestingly, ASTs, EPNs, GLUTNs, OPCs, and PERs showed the highest number of DEGs, while macrophages showed almost no changes in DEGs. We also identified cell type–specific DEGs, e.g., *Ler2* for ASTs, *Fth1* enriched in ECs, *Cnr1* and *Sox4* in GABANs, and *Reln* for GLUTNs.

We found that more than 30% DEGs of glutamatergic neurons in the cAMP signaling pathway belonged to the ATPase plasma membrane Ca^2+^ transporting family. Previous research has demonstrated that when Ca^2+^ signaling is abnormal, a balance in activities of Ca^2+^-calmodulin-dependent kinase II and Ca^2+^-dependent phosphatase calcineurin is shifted at the synapse, tilting the balance between long-term potentiation (LTP) and long-term depression (LTD) synaptic mechanisms. As a result, synapses are weakened and eliminated by LTD mechanisms, causing memory loss [55]. Furthermore, C1qb was increased in glutamatergic neurons after 2VO surgery. C1qb immunoreactivity was detected in hippocampal GLUTNs as small punctate deposits in the hippocampus after kainite injection. It was found that neuronal-derived C1q plays a critical role in synaptic refinement. Although microglia express higher levels of C1q than neurons, several lines of evidence suggest that neurons also contribute significantly to C1q levels and complement-dependent synaptic refinement [56]. All these results strongly implied that C1qb is a potential therapeutic target for post-ischemia memory impairment.

Intriguingly, *Lars2* was also the most significantly decreased gene in GABANs after 2VO surgery (Fig. 3E, right and Supplementary Table 6), whereas *Cnr1* was the most significantly increased gene. CNR1 is located in the presynaptic terminals of the prefrontal, temporal, and hippocampal regions and exhibits numerous interactions with various central neurotransmitters [57, 58]. Research has found a significant association between CNR1 and neurocognition [59]. Specifically, attention and working memory performance have been shown to be compromised under acute effects of the CNR1 agonist, 9-tetrahydrocannabinol [60]. However, the exact mechanisms in ischemic complications are still not completely understood.

Several genes mainly expressed in microglia were upregulated in astrocytes (Fig. 4E). *C1q* knockout mice were significantly protected against hypoxic-ischemic brain injury and exhibited significantly less neurofunctional deficit compared with wild-type mice [61]. Consistent with our results, *C1q* mRNA has also been reported to be dramatically upregulated in the brain after global ischemia and focal ischemic insult in rodents [44]. Recent research found that *Hexb* was persistently expressed by microglia and much less by central nervous system–associated macrophages in various disease models. All those results implied that astrocytes functions as the microglia-like cell type after 2VO surgery.

Neurogenesis is the process of producing new functional neurons from other cell types, including proliferation and differentiation of progenitor cells into mature neurons. In cerebral ischemia, enhanced neurogenesis has been reported after stroke [62], suggesting a potential avenue for ischemia therapy. OPCs are multipotential cells and able to differentiate into other cell types including OLGs and neurons [48]. Consistent with previous reports [63], we identified four OPCs subtypes (Fig. 5B) with possible markers for each subtype. Slingshot pseudotime analysis showed developmental trajectory from OPC-4 to OPC-1, then to OPC-2 and finally to OPC-3 (Fig. 5I), suggesting OPC-4 is the primary cluster while OPC-3 was defined as a cluster that is committed to differentiation into OLGs. OPC-1 (Cspg5 + OPCs) was found to highly express the immature neuronal marker *Sox11*. Cspg5, originally found in the developing brain, is likely involved in neurogenesis and synaptogenesis. High expression of Cspg5 in one of the subclusters of OPC has been described recently based on single-cell sequencing analysis either [64]. *Vcan*, mainly expressed in OPC-2, also named *Cspg2*, is involved in cell proliferation and a major component of the extracellular matrix [65]. *Fyn*, mainly expressed in OPC-3, is required for cell-cycle progression through G1 phase, plays important role in cell growth [66]. *Pclaf*, mainly expressed in OPC-4, also named as *KIAA0101*, acted as a regulator of DNA repair during DNA replication, has been reported to be crucial for cell proliferation [50, 51]. Together, all those data suggested that OPCs were promising neurons-generated cell type for neurogenesis.

*Lars2* was also one of the most significantly decreased genes in the 2VO group of ECs. Lars2 plays vital roles in catalyzing the aminoacylation of tRNA^Leu^. Interestingly, the most upregulated DEG in ECs was *Ttr* (Supplementary Table 12). Ttr is a thyroid hormone–binding protein, transporting thyroxine from the bloodstream to the brain [67]. In addition, we found that *plp1*, a major component of mammalian CNS myelin with possible functions in myelin stability and maintenance, was sharply increased in ECs after ischemia (Supplementary Table 12). However, the role of Plp1 in ECs after 2VO surgery needs to be further explored. The results showed that DEGs in ECs were mainly enriched in the protein processing pathway of the endoplasmic reticulum (Fig. 6F). Similar with our results, it has been shown that foldases encoding genes were increased in the endoplasmic reticulum on days 7 and 14 after ischemia [68].

Lcn2, also called neutrophil gelatinase–associated lipocalin or 24p3, is a crucial component of neutrophils [69]. Lcn2 participates in innate immune responses, impacts on cell proliferation and differentiation, and regulates iron homeostasis [70]. It has been reported that Lcn2 is expressed in the ischemic brain after temporary experimental ischemia and accompanied by the accumulation of cellular nonheme iron. Plasma levels of Lcn2 measured in patients 1 week after ischemia contribute to the prediction of clinical outcome at 90 days and reflect the systemic response to post-ischemia infections. Therefore, Lcn2 is likely to have value as a biomarker in ischemia patients [71]. Similar with our results, previous studies have shown that Lcn2 is secreted in response to cerebral ischemia to promote reperfusion injury. Genetic deletion of Lcn2 significantly reduced brain injury after cerebral ischemia, implying that Lcn2 is a mediator of reperfusion injury and a potential therapeutic target. Immunotherapy has the potential to harness neuroinflammatory responses and provides neuroprotection against ischemia. Research has found that Lcn2 is induced on the inner surface of the cerebral ECs. Its monoclonal antibody was found to specifically target Lcn2 in vitro and in vivo, attenuating the induction of Lcn2 and pro-inflammatory mediators after ischemia. Administration of Lcn2 antibody at 4 h after ischemia significantly reduced neurological deficits, cerebral infarction, edema, blood brain barrier leakage, and infiltration of neutrophils. The binding epitope of Lcn2 antibody was mapped to its β3 and β4 strands, which are responsible for maintaining the integrity of the Lcn2 cup-shaped structure [72], indicating that Lcn2 can be pharmacologically targeted using a specific antibody to reduce reperfusion injury after ischemia and suggesting that early detection and inhibition of Lcn2 may prove useful in the diagnosis and treatment of post-ischemia memory impairment.

*Apoe*, the most significantly increased DEG in microglia after 2VO surgery (Fig. 7E, above), is associated with age-related risk for Alzheimer’s disease and plays critical roles in Aβ homeostasis. S100B, which is highly expressed in cluster 8 of OLGs, is a 21 kDa Ca^2+^ binding protein that was reported to be expressed in Schwann cells and astroglia. It has often been reported as a promising biomarker for ischemia [73]. *S100b* has also been detected in numerous other cell types in the CNS, such as OLGs, ependymal cells, the choroid plexus epithelium, and neurons [74]. However, the function of S100b in OLGs needs to be further explored after cerebral ischemia. *Neat1*, the most significantly upregulated DEG in OLGs after 2VO surgery (Fig. 8H, Supplementary Table 18), is a long noncoding RNA that is essential for the formation of paraspeckles and interacts with many intracellular regulatory factors that has been widely investigated in the cancer field [75]. *Cnp* was the most significantly decreased DEG in OLGs (Fig. 8H, Supplementary Table 18). The absence of *Cnp* was found to be associated with an impaired cellular transport in the axonal compartment [76]. Taken together, the results indicate that *Cnp* plays critical roles in the regulation of memory, and increasing the expression of *Cnp* in OLGs may be a potential therapy in the treatment of ischemia.

In single-cell transcriptome, each cell needs to be isolated from the living tissues. The morphological complexity of cells like those of the central nervous system makes the segregation process very difficult. The separation process exposes them to specific environmental, chemical, and harsh dissociation steps which often bias data analysis. In addition, it is very hard to obtain the healthy human brain tissue required by the single-cell transcriptome, so most of the single-cell sequencing is conducted in animals at present, but the extent to which they can represent the human brain tissue is unknown. Though 10 × Genomics single-cell sequencing platform allows analysis of cells in an unbiased manner, it lacks in providing an in-depth information on sequence diversity, splicing, and chimeric transcripts generated in the process [77]. Furthermore, when the cells are dissociated or isolated, a certain number of cells become dead or get destroyed. The single-cell RNA sequencing methods generate low-quality data from these cells. And many transcripts appear to be lost during reverse transcription due to the small number and low capture efficiency of RNA molecules in single cells [78]. Of note, our cell sampling strategy resulted in biases in cell proportions between control and 2VO groups based on the RxC analysis results. In addition to distinctness in the original composition ratio, differences in the sensitivity of cells, such as neurons, to external stimuli can cause this situation. Besides, environmental, chemical, and harsh dissociation steps may result in the shift of cell proportions, and even affect the results of GSEA and DEG results between control and 2VO groups Finally, in this study, conclusions are based on the minimal number of biological replicates (*n* = 2 for each group); further research is needed to determine whether the results are universal. Meanwhile, we only studied the gene expression changes of different types of cells in the hippocampus on the seventh day after ischemia, which does not fully reflect the trend and rules of gene changes with time after ischemia.

Although there is much more to explore further, this study reveals neuronal, glial, and vascular lineage heterogeneity and cell-specific molecular changes in post-ischemia memory impairment. The results of our study will help to advance a variety of efforts towards understanding the function of different cell types and exploring molecular and cellular therapeutic targets for post-ischemia memory impairment.

### Supplementary Information

Below is the link to the electronic supplementary material.

Supplementary file1 (XLS 543 KB)

Supplementary file2 (XLSX 9.92 KB)

Supplementary file3 (XLSX 10 KB) Cell numbers in each neuronal subcluster of each sample;

Supplementary file4 (XLSX 307 KB) High expressed genes in each neuronal subcluster;

Supplementary file5 (XLS 17 KB) The differential expressed genes of glutamatergic neurons after PSCI treatment;

Supplementary file6 (XLS 29 KB) The differential expressed genes of GABAergic neurons after PSCI treatment;

Supplementary file7 (XLS 23 KB) The KEGG analyzed results in astrocytes after PSCI treatment;

Supplementary file8 (XLS 17 KB) The differential expressed genes in astrocytes after PSCI treatment;

Supplementary file9 (XLS 17 KB) The differential expressed genes in oligodendrocyte precursor cells after PSCI treatment;

Supplementary file10 (XLS 40 KB) The GO analyzed results in oligodendrocyte precursor cells after PSCI treatment;

Supplementary file11 (XLSX 22 KB) The differential expressed genes in OPC-4 after PSCI treatment;

Supplementary file12 (XLS 5 KB) The differential expressed genes in endothelial cells after PSCI treatment;

Supplementary file13 (XLSX 19 KB) The differential expressed genes in subcluster 3 or 7 of endothelial cells after PSCI treatment;

Supplementary file14 (XLSX 19 KB) The differential expressed genes in subcluster 2 or 12 of endothelial cells after PSCI treatment;

Supplementary file15 (XLS 5 KB) The differential expressed genes in subcluster 20 of endothelial cells after Supplementary PSCI treatment;

Supplementary file16 (XLSX 98 KB) The differential expressed genes of immune cells after PSCI treatment;

Supplementary file17 (XLSX 144 KB) High expressed genes in each immune cell subcluster;

Supplementary file18 (XLS 7 KB) The differential expressed genes of oligodendrocytes after PSCI treatment;

Supplementary file19 (XLS 22 KB) The differential expressed genes of ependymocytes after PSCI treatment.

Supplementary file20 (DOCX 48 KB) Cell counts in Sham and 2VO groups of different cell types.

Supplementary file21 (DOCX 41 KB) Cell proportions (%) in Sham and 2VO groups of different cell types.

Supplementary file22 (DOCX 64 KB) Statistics of 95% CI for proportion of sham in each cell type.

Supplementary file23 (PDF 9.16 MB)

## Data Availability

The single-cell RNA sequencing data are available through NCBI's Gene Expression Omnibus (https://www.ncbi.nlm.nih.gov/geo/) under the accession number GSE 171393.

## Abbreviations

2VO: Two-vessel occlusion
SPF: Specific pathogen free
GEM: Gel bead in emulsion
PCA: Principal component analysis
t-SNE: T-Distributed stochastic neighbor embedding
UMAP: Uniform manifold approximation and projection
GSEA: Gene set enrichment analysis
NES: Normalized enrichment score
PBS: Phosphate-buffered saline
PFA: Paraformaldehyde
OCT: Optimal cutting temperature compound
ANOVA: Analysis of variance
RM: Repeated measured
CI: Confidence interval
OLG: Oligodendrocyte
AST: Astrocyte
OPC: Oligodendrocyte precursor cell
NPC: Neuronal precursor cell
GLUTN: Glutamatergic neuron
GABAN: GABAergic neuron
EPN: Ependymocyte
CPEC: Choroid plexus epithelial cell
EC: Endothelial cell
PER: Pericyte
VSMC: Vascular smooth muscle cell
VLMC: Vacuolar leptomeningeal cell
KEGG: Kyoto Encyclopedia of Genes and Genomes
GO: Gene ontology
DEG: Differentially expressed gene
CNS: Central nervous system
LTP: Long-term potentiation
LTD: Long-term depression
