# Supplementary material for: Single-Cell Transcriptome Reveals Cell Type–Specific Molecular Pathology in a 2VO Cerebral Ischemic Mouse Model
Source: Mol Neurobiol. 2024 Jan 5;61(8):5248–64. doi: 10.1007/s12035-023-03755-4 (PMC11249492; doi:10.1007/s12035-023-03755-4)
Supplement: Supplementary file 20 — Supplementary file20 (DOCX 48 KB) Cell counts in Sham and 2VO groups of different cell types. [file 12035_2023_3755_MOESM20_ESM.docx]

**Supplementary Table 20 Cell counts in Sham and 2VO groups of different cell types.**
