# Supplementary figures and images for: Single-Cell Transcriptome Reveals Cell Type–Specific Molecular Pathology in a 2VO Cerebral Ischemic Mouse Model
Source: Mol Neurobiol. 2024 Jan 5;61(8):5248–64. doi: 10.1007/s12035-023-03755-4 (PMC11249492; doi:10.1007/s12035-023-03755-4)

**A****DAPI****SMI-32****MBP****Merge****Sham**

CTX

CC

STR

**2VO**

50um

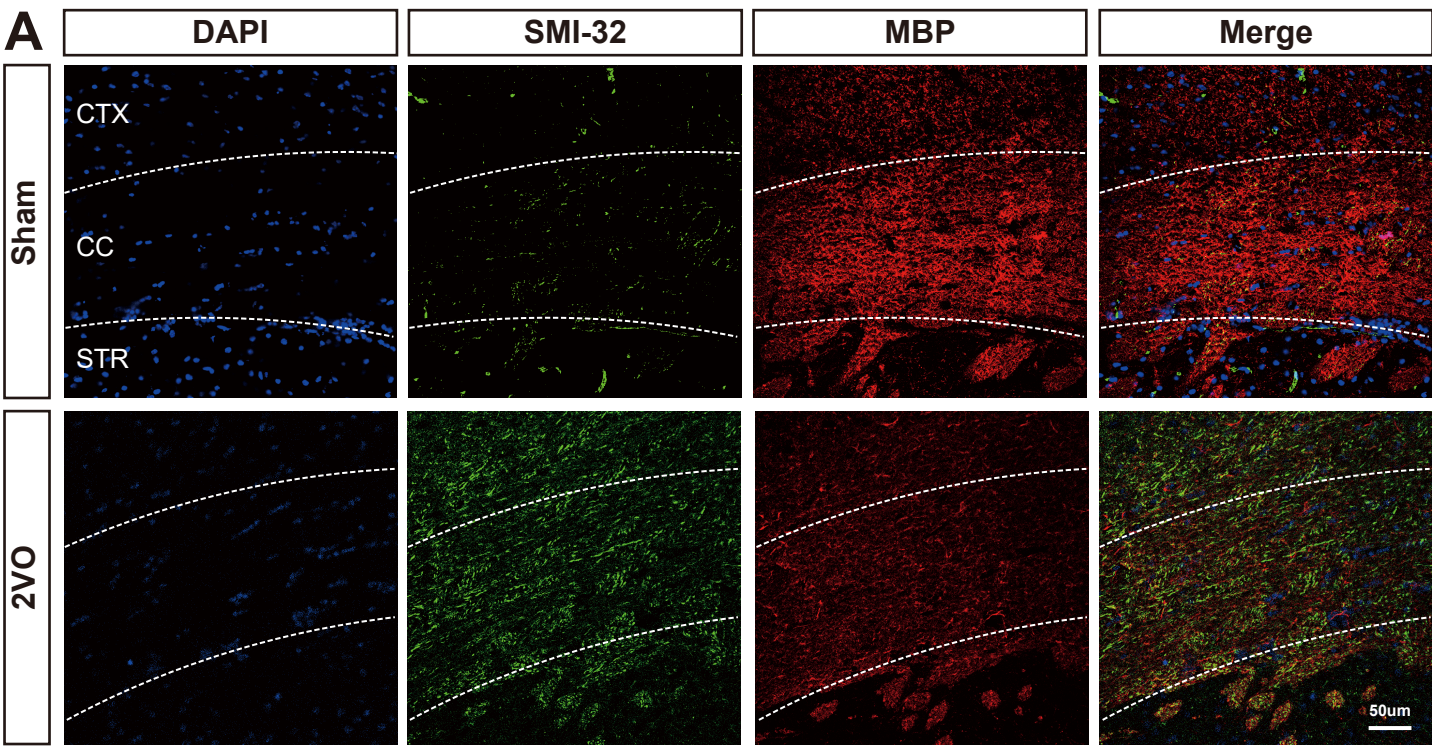

Supplement: Supplementary file 23 — Supplementary file23 (PDF 9.16 MB) [file 12035_2023_3755_MOESM23_ESM.pdf]
